# Development of a parent version of the Manchester-Minneapolis quality of life survey for use by parents and carers of UK children: MMQL-UK (PF)

**DOI:** 10.1186/1477-7525-6-19

**Published:** 2008-02-28

**Authors:** Hayley A Hutchings, Penney Upton, Wai-Yee Cheung, Alison Maddocks, Christine Eiser, John G Williams, Ian T Russell, Sonia Jackson, Meriel EM Jenney

**Affiliations:** 1 School of Medicine, Swansea University, Swansea, UK; 2 Institute of Health, Social Care and Psychology, University of Worcester, Worcester, UK; 3 National Public Health Services for Wales, Carmarthen, UK; 4 Child and Family Research Group, Department of Psychology, University of Sheffield, Sheffield, UK; 5 Institute for Medical and Social Care Research, University of Wales Bangor, Bangor, UK; 6 Thomas Coram Research Unit, Institute of Education, University of London, London, UK; 7 Department of Child Health, Cardiff and Vale NHS Trust, Cardiff, UK

## Abstract

**Background:**

Although it is now widely endorsed that children should as far as possible rate their own health related quality of life (HRQL), there are situations where proxy information on child HRQL may be useful, especially where a child is too ill or young to provide their own HRQL assessment. There is limited availability of generic HRQL scales that have a parallel child and parent version and that are reliable, valid, brief, comprehensible and suitable for use in UK populations. The aims of this study were therefore to develop and validate a parent version of the anglicised Manchester-Minneapolis Quality of Life child form (MMQL-UK (CF)) and to determine the level of association between the child and parent versions of this form.

**Methods:**

This study was undertaken concurrently with the anglicisation and validation of the MMQL, a measure of HRQL developed for use with children in North America. At that time, no parent version existed, so the MMQL form for children (MMQL-UK (CF)) was used as the basis for the development of the MMQL-UK parent form (PF). The sample included a control group of healthy children and their parents and five exemplar groups; children diagnosed with asthma, diabetes or inflammatory bowel disease and their parents, children in remission from cancer and their parents and children in public care and their carers. Consistency of the MMQL-UK (PF) components were assessed by calculating Cronbach's alpha. Validation of the parent questionnaire was undertaken by comparing MMQL-UK (PF) component scores with comparable components on the proxy PedsQL™ quality of life scales, comparing MMQL-UK (PF) component scores between parents of healthy and chronic disease children and by comparison of component scores from children and their parents or carers. Reproducibility and responsiveness were assessed by retesting parents by follow-up questionnaires.

**Results:**

A total of 874 children (completing MMQL-UK (CF)) and 572 parents or carers (completing MMQL-UK (PF)) took part in the study. The internal consistency of all the MMQL-UK (PF) components exceeding the accepted criterion of 0.70 and the construct validity was good with moderate correlations being evident between comparable components of the MMQL-UK (PF) and the proxy PedsQL™. Discriminant validity was demonstrated with significant differences being identified between parents of healthy children and those with chronic conditions. Intra-class correlations exceeded 0.65 for all MMQL-UK (PF) components demonstrating good reproducibility. Weak to moderate levels of responsiveness were demonstrated for all but social functioning. The MMQL-UK (PF) showed moderate parent-child correlation with the MMQL-UK (CF) for all components. The best correlations were seen for those components measuring the same construct (Pearson's r ranged from 0.31 to 0.61, p < 0.01 for equivalent components).

**Conclusion:**

The MMQL-UK (PF) showed moderate to good correlations with the MMQL-UK (CF) component scores. The MMQL-UK (PF) will be of use when comparing child and parent/carer perception of the impact of a child's condition on their HRQL or where the child is too ill or young to provide their own report.

## Background

The measurement of health-related quality of life (HRQL) is increasingly becoming part of the overall assessment of a patient's health both in the clinical and the research setting, as it provides a more complete picture of the health of the patient. Measuring HRQL includes assessing the patient's perceptions of their physical, emotional and social health and function that can supplement clinical information on their health status.

It is now widely endorsed that children as far as possible should rate their own HRQL [1,2]. However, there is some suggestion that children may not be able to provide accurate and reliable assessments of their HRQL [3] and that this may be due to problems understanding the questions, lack of understanding of the disease itself or time perception differences [4]. Assessment of HRQL in children may also be compromised by their age and development and by their ability to complete lengthy questionnaires [5].

There are conflicting views regarding the benefits of using proxies for measuring HRQL. There is some doubt as to whether proxy reports provide an accurate reflection of the child's HRQL [3,6-8]. Patient and proxy reports may differ because of the lack of parallel content in the instruments used [9]. Some studies have highlighted limited concordance between child and parent reports [9-12] whilst others have reported more complementary information from children and their parents [7,13]. What is important about proxy ratings is understanding when parents are able to provide useful information. This is more likely to be in relation to impact of HRQL on the family, sibling relationships and to a lesser extent school progress [14]. Parents' knowledge of their child is likely to be more limited in relation to activities or relationships that exist outside home and with respect to internal feeling states [9,10,12]. Parents also have a role to play when a child is too young or ill to provide a HRQL assessment. It has been suggested that consistent parent-proxy reports may prove to be more reliable and valid in longitudinal HRQL and long-term outcome investigations than their children's own reports alone because of the rapid changes in children's attitudes, abilities, and priorities as part of the normal developmental process [15]. The solution is to regard both child and parental assessment as valid and contributing to the total picture regarding the child's HRQL [14].

When measuring the HRQL of children it may be necessary or desirable to obtain reports from their parents [10]. Clinicians often look to parents for guidance regarding their child's HRQL as they play an important role in medical decision making [16]. It is important that the clinician is confident in a parent's evaluation of their child's HRQL. If a child is unable to provide HRQL judgements, drawing on alternate sources of information regarding a patient's HRQL may be a useful source of information provided that additional information is available to support the validity of the proxy ratings [16]. Even when a child's responses are available, the perspective of the parent has an important bearing on health care decisions with respect to the child. For these reasons, parallel reporting is increasingly recommended in studies involving the assessment of health outcomes in child populations with analogous questionnaires for children and their parents being developed [10].

In spite of this, may measures of child HRQL do not include parallel parent versions [17,18]. For this reason, we report the development and validation of a parent version of the Minneapolis-Manchester Quality of Life Instrument (MMQL-UK (PF)) that could be used in parallel with the child version to assess the HRQL of children with chronic conditions, those in public care and their healthy peers.

## Methods

### Study questionnaires

#### Minneapolis-Manchester Quality of Life Instrument (MMQL)

This questionnaire was originally developed in the US for use with child cancer survivors [19]. The original version of the MMQL had three different forms, one for children aged 8–11 (Child form, MMQL-CF), one for 12–18 year olds (Youth Form, MMQL-YF) and one for young people aged 19–25 years. All the original child MMQL forms measured the components of physical functioning, psychological functioning, social functioning, cognitive functioning, body image and outlook on life. Intimate relations was an additional component in the questionnaires for 12–18 and 19–25 year olds. No parent form is available for the original MMQL forms. The anglicised MMQL-UK (CF) version of the questionnaires (based on the 12–18 year age group) was used as the basis for the development of the parent form (MMQL-UK (PF)). This measured the components of physical appearance, school functioning, social functioning, emotional functioning and physical functioning [20].

#### PedsQL™ version 4.0 core module

PedsQL™ measurement system is a modular approach to measuring HRQL in children and adolescents and their parents which is rapidly becoming established in the US and Europe [21,22]. Anglicised versions are also available [13]. It consists of a brief, practical generic core module, which is complemented by a number of condition specific measures. Reliability and validity of the generic core module has been demonstrated [22]. PedsQL™ measures the components of physical functioning, emotional functioning, social functioning and school functioning. Parents or carers were asked to complete this core module as part of the validation of the newly developed parent MMQL-UK.

### Study population

This study was carried out in the context of a wider study to anglicise and shorten the Manchester-Minneapolis Quality of Life Survey for children (MMQL-UK (CF))[20]. The MMQL-UK (CF) was used as the basis for the development of an adult form (MMQL-UK (PF)).

Before wider administration of the MMQL-UK (PF) could take place, the phrasing of the questions from the child form had to be modified to make them adult oriented. This was done by qualitative methods. An opportunistic sample of 15 parents of school children (age range 10–18) were recruited for interviews from a local school known to one of the researchers (PU) for development of the MMQL-UK (PF). During the interviews parents were asked to comment on the wording and structure of the questionnaire as well as completing a draft parent form. Interviews were carried out either at the home or at Swansea University, depending on the preference of the parent.

The newly developed MMQL-UK (PF) was then compared with the shortened and anglicised version of the MMQL-UK (CF). This paper concentrates specifically on the validation of the MMQL-UK (PF) and comparison with the child (CF) version. The Anglicisation and validation of the MMQL-UK (CF) is reported elsewhere [20]. Although the original MMQL form was developed to measure HRQL in cancer survivors the anglicised MMQL-UK (CF) was developed for use with both healthy and chronic conditions. The MMQL-UK (PF) was therefore developed to be suitable for the same populations (i.e. healthy and chronic conditions).

Children and their parents or carers who met the inclusion criteria (see Table 1) were approached to take part in the study comparing the child and parent MMQL. Four chronic conditions (asthma, diabetes, chronic inflammatory bowel disease (IBD) and allogenic bone marrow transplant (BMT) following acute lymphoblastic leukaemia (ALL)) were chosen as exemplars for the study. In addition 'looked after' children in public care were recruited as a fifth exemplar. These groups were chosen to ensure a cross section of conditions varying in chronicity and degree of self-care involved and for their diverse impact on the different domains of childhood HRQL. Children with a chronic health problem were identified with the guidance of collaborating clinicians. Children in public care were identified through looked after assessments.

**Table 1:** Inclusion criteria for children and parents entering the study

Group	Inclusion criteria
Asthma	Children aged 8–18 years with moderate/severe asthma according to definitions given by the British Thoracic Society and their parents
Diabetes	Children aged 8–18 with Type 1 Diabetes Mellitus and their parents
IBD	Children aged 8–18 fulfilling diagnostic criteria for Crohn's Disease and Ulcerative Colitis and their parents
BMT	Children aged 8–18 years at least six months post treatment for ALL and their parents
Public care	Children aged 8–18 years in public care and their carers
Controls	Healthy children aged 8–18 years from local schools

Where possible, written information was sent out to the families of children with a chronic health condition one week before a routine outpatient appointment. On attendance at the clinic, a researcher provided a further verbal explanation of the study, took written consent and supervised completion of the questionnaires. Families of children not due in clinic during the data collection period were sent written information along with a reply form on which they could indicate their interest in the study. Telephone contact was made with families responding positively, to provide further verbal feedback about the study. Arrangements were made for families agreeing to take part to complete the questionnaire either in clinic or at home. Children in public care and their carers were provided with verbal and written information when attending paediatric assessments. If families expressed an interest in the study, arrangements were made for a home visit, where the interviewer gained informed consent and questionnaires were completed.

Participants for the control group were recruited from schools in Swansea, Neath, Port Talbot and Bridgend (Wales, UK). The control group were healthy children (and their parents) who were not currently using health care resources for any serious condition. Participants were given a screening questionnaire prior to participation that included a section about health status. Anyone who was identified as having a health problem was excluded from participation in the study. Permission to approach schools was obtained from the Directors of Education in each Local Education Authority, before selecting schools via multi-stage sampling. Information was sent to head teachers of sampled schools, inviting their school to participate. A researcher visited schools expressing an interest, to discuss the project further and arrange for data collection. Written information for parents, parental consent forms and parent questionnaires were supplied for each child to take home. Parents were asked to complete their questionnaires at home and return them to school by a specified date, along with consent for their child to complete the questionnaires. It was emphasised that completion of questionnaires should not involve consultation with the child. For those children that had parental consent, re-explanation and completion of questionnaires followed in class a week later, under the supervision of the researcher.

In both subject and control groups, children with moderate to severe learning difficulties and children and parents for whom English was not their first language were excluded from the study.

The study was approved by the Welsh Multi-centre Research Ethics Committee (MREC). Informed consent was sought from all children (subjects and controls) and their parents or those with parental responsibilities (for children less than 16 years of age), following oral and written explanation of the study.

### Analysis

Data were analysed using the Statistical Package for Social Sciences (SPSS) version 11.4.

#### Assessing internal consistency

The internal consistency of the MMQL-UK (PF) components were assessed by item-total correlations and Cronbach's alpha [23]. Questions yielding item-total correlations below 0.4 were considered for rejection [24]. Questions were also considered for rejection if more than 75% of individuals gave the same response, because such questions are not sensitive enough to discriminate between different levels of severity [24]. Questions were also considered for exclusion if they were disliked or considered difficult to answer by the parents completing them. Cronbach's alpha for each of the resulting components should exceed 0.7 [25]. Principal components analysis was then performed on the parent content without any restrictions on the data and the structure was compared to the emergent structure from the child form. This was carried out in order to validate the underlying components of the MMQL-UK (PF).

#### Assessing validity

The construct validity of the MMQL-UK (PF) components were assessed by comparing them with the appropriate parent PedsQL™ quality of life scales. If the components were valid measures of HRQL, they would be expected to show significant small to moderate levels of correlation with each of the PedsQL™ scales, with the largest correlations being seen between the PedsQL™ scales measuring physical, social, emotional and school function and the comparable MMQL-UK (PF) components.

The discriminant validity of the MMQL-UK (PF) was assessed by comparison of component scores between parents whose children were being treated and the parents of the control group. Our a priori hypothesis was based on identifying moderate to large differences in effect size between each exemplar and the control group [26]. If the components were valid measures of HRQL, the exemplar groups would be expected to score lower on the MMQL-UK (PF) components than the control group. Independent samples t-tests with Bonferroni corrections were used to compare control and exemplar groups. Differences in component score between the exemplars and control groups were also reported as effect sizes where 0.2–0.49 represented a small difference, 0.5–0.79 represented a moderate difference and greater than 0.8 represented a large difference [26].

The relationship between the MMQL-UK (CF) and MMQL-UK (PF) was determined using Pearson's correlation. If the two reports measure the same concept, then moderate correlation would be expected between the component scores of the MMQL-UK (CF) and the MMQL-UK (PF).

#### Assessing reproducibility

Following initial completion of the MMQL-UK (PF) by parents of the exemplar group children, they were asked to complete a second 'retest' questionnaire the next time their child visited clinic. This was a maximum of three months after the first assessment. In addition to completing the MMQL-UK (PF), the parents of children were asked to rate whether they thought their child's health had changed (improved, got worse or stayed the same) since the first questionnaire was completed. Those parents reporting no change were included in the reproducibility analysis. Reproducibility was assessed using intra-class correlation co-efficient [27].

#### Assessing responsiveness

The responsiveness of the MMQL-UK (PF) was assessed by using the scores of the exemplar group parents who reported a change in their child's health. The response ratio (mean change in scores for subjects reporting a change divided by the standard deviation of the subjects reporting no change) was used to quantify the responsiveness [27]. The larger the ratio, the more responsive the instrument.

## Results

In order to have a parallel proxy report, question selection for the parent form replicated that for the child form. A total of 29 questions were selected for the anglicised and shortened MMQL-UK (CF) and MMQL-UK (PF) with five proposed components: physical appearance, school functioning, social functioning, emotional functioning and physical functioning.

874 children and 572 parents completed the MMQL-UK (CF) and MMQL-UK (PF). The internal consistency of the MMQL-UK (PF) was excellent, with all components exceeding the accepted criterion of 0.70 (see Table 2)

**Table 2:** Internal consistency of the MMQL-UK (PF) component

Scale	Minimum corrected item- component correlation	Maximum corrected item-component correlation	Cronbach's alpha
Physical Appearance	0.53	0.71	0.90
School Functioning	0.37	0.83	0.93
Social Functioning	0.64	0.78	0.90
Emotional Functioning	0.35	0.56	0.75
Physical Functioning	0.26	0.64	0.77

Minimum and maximum values represent the lowest and highest scores for the individual components of each of the sub-scales.

The construct validity of the MMQL-UK (PF) was good, with significant correlations being evident between the MMQL-UK (PF) and the proxy PedsQL™ on those scales measuring similar constructs, i.e physical functioning, emotional functioning, social functioning and school functioning (see Table 3).

**Table 3:** Correlations between the MMQL-UK (PF) and the PedsQL™ proxy-report components

	MMQL-UK (PF)				
PedsQL™ Proxy-report	Physical Appearance	School Functioning	Social Functioning	Emotional Functioning	Physical Functioning

Physical Functioning	0.27**	0.19**	0.28**	0.23**	0.34**
Emotional Functioning	0.28**	0.25**	0.36**	0.61**	0.19**
Social Functioning	0.34**	0.30**	0.50**	0.41**	0.40**
School Functioning	0.17**	0.58**	0.29**	0.24**	0.28**

** Significant at the 0.01 level

Table 4 illustrates significant differences between the mean component scores for the parents of control and exemplar groups. All exemplar proxies reported lower HRQL for physical functioning than controls. Proxy physical appearance scores were significantly lower for the diabetes, cancer and IBD groups compared with controls and proxy emotional functioning reported by parents was lower for all children with a chronic health problem when compared with controls. Proxy school functioning scores were significantly lower compared with controls for the cancer group and those in public care and proxy social functioning was significantly lower for the cancer group and those in public care than for controls.

**Table 4:** Mean scores (Standard Deviation) for the MMQL-UK (PF) components for the different patient groups and controls

MMQL-UK (PF)	Control (n = 296)	Asthma (n = 37)	Diabetes (n = 70)	Cancer (n = 42)	IBD (n = 56)	Public Care (n = 71)
Physical Appearance	76.9 (22.26)	64.9 (31.8) ES 0.54	67.7 (25.3)* ES 0.50	56.8 (23.6)** ES 0.99	59.5 (25.8)** ES 0.83	69.5 (25.8) ES 0.42
School Functioning	76.2 (26.0)	73.1 (26.5) ES 0.11	73.4 (25.7) ES 0.11	45.2 (33.9)** ES 1.02	68.8 (26.0) ES 0.28	52.0 (32.0)** ES 0.83
Social Functioning	86.4 (16.1)	84.6 (20.3) ES 0.10	87.1 (16.6) ES -0.04	78.3 (16.9)* ES 0.50	83.0 (16.4) ES 0.21	73.5 (25.0)** ES 0.61
Emotional Functioning	69.1 (11.6)	63.1 (13.9)* ES = 0.47	62.1 (13.3)** ES = 0.56	60.7 (11.3)** ES = 0.73	59.7 (16.6)** ES = 0.66	67.5 (18.8) ES = 0.10
Physical Functioning	86.8 (16.8)	56.5 (28.0)** ES = 1.31	80.4 (18.0)* ES = 0.37	55.6 (29.5)** ES = 1.30	55.9 (27.8)** ES = 1.28	78.2 (20.7)** ES = 0.46

Independent samples t-test with Bonferroni correction for multiple comparisons.

** Significant at the 0.01 level.

*Significant at the 0.05 level.

ES = effect size: small = 0.2–0.49; moderate = 0.5–0.79; large>0.8 [26]

Intra-class correlations ranged from 0.65–0.91 for the MMQL-UK (PF) components demonstrating good reproducibility for the questionnaire (see Table 5)

**Table 5:** Reproducibility of the MMQL-UK (PF) components for parents reporting no change in their child's health status (N = 50)

Component	Mean difference (SD) retest-test	95% CI for the difference	Intraclass correlation
Physical Appearance	1.40 (17.76)	-3.65 to 6.45	0.77
School Functioning	-0.07 (16.44)	-4.79 to 4.65	0.84
Social Functioning	-1.50 (15.38)	-5.87 to 2.87	0.65
Emotional Functioning	0.35 (12.61)	-3.24 to 3.93	0.75
Physical Functioning	-0.16 (11.76)	-3.44 to 3.24	0.91

Weak to moderate levels of responsiveness were demonstrated for all components except social functioning, with ratios for emotional and physical functioning reaching significance. The negative ratio for social functioning however, suggested that reported changes in HRQL were in the opposite direction to changes in health status (see Table 6)

**Table 6:** Responsiveness of the MMQL-UK (PF) components for parents reporting an improvement or deterioration in their child's health status

Scale	Mean difference for subjects reporting a change (n = 19)	Two tailed significance	SD of the scores of the stable subjects (n = 50)	Responsiveness ratio
Physical Appearance	5.00	0.15	17.76	0.28
School Functioning	4.70	0.207	16.44	0.29
Social Functioning	-0.66	0.67	15.38	-0.04
Emotional Functioning	7.71	0.003**	12.61	0.61
Physical Functioning	12.63	0.017*	11.76	1.07

** Significant at the 0.01 level.

*Significant at the 0.05 level

Table 7 illustrates the correlations between the MMQL-UK (CF) and the MMQL-UK (PF) for all the controls and exemplar groups. The individual exemplar groups were examined in addition to the whole group to determine if the correlation patterns were similar. We were particularly interested in the looked after children group as the proxy form was completed by the carer as opposed to the parents as in the other exemplar groups. All the groups showed similar correlations and as such the data were analysed as one group. Statistically significant moderate to good correlations were seen for all five components between the MMQL-UK (CF) and the MMQL-UK (PF).

**Table 7:** Correlations between the MMQL-UK (CF) and MMQL-UK (PF)

	MMQL-UK (PF) component				
MMQL-UK (CF) component	Physical Appearance	School	Social Functioning	Emotional Functioning	Physical Functioning

Physical Appearance	0.51**	0.05	0.20**	0.25**	0.12**
School	0.13**	0.56**	0.22*	0.14**	0.21**
Social Functioning	0.14**	0.10*	0.31**	0.23**	0.18**
Emotional Functioning	0.35**	0.20**	0.26**	0.38**	0.25**
Physical Activities	0.33**	0.25**	0.21**	0.27**	0.61**

** Significant at the 0.01 level.

*Significant at the 0.05 level

## Discussion

A parent version of the MMQL (MMQL-UK (PF)) was successfully developed based on the anglicised and shortened child version which has been recently validated in the UK (MMQL-UK (CF))[20]. The parent version was developed in parallel with the child version with only minor modifications being made to the wording of the questions.

Good internal reliability was found for the MMQL-UK (PF), with alphas exceeding 0.70 for all the components, thus making them acceptable for group comparisons [25]. Construct validity of the MMQL-UK (PF) was established through moderate correlations with the parent PedsQL™ core module.

Discriminant validity was demonstrated for the MMQL-UK (PF) proxy report with differences being noted in HRQL by parents of healthy children and children with chronic health conditions and carers of children in local authority care. The reproducibility and responsiveness of the MMQL-UK (PF) were indicated by good test-retest results. The relationship between the MMQL-UK (CF) and MMQL-UK (PF) was confirmed by moderate correlations indicating that the two reports measure the same concept.

The availability of a proxy report is important as there may be occasions when a child is either unable or unwilling to complete a HRQL measure. However interpretation of proxy reports should be undertaken with caution as they may not accurately reflect the child's view, particularly for some components. However if they are used in conjunction with a clinical consultation or other objective assessments they could potentially provide useful information on the health of the child [16].

It should be recognised that both child and proxy ratings have value but the question is to clarify how differences in perception of HRQL arise between the child and the proxy [5]. It has been contested that disagreement is not necessarily undesirable and multiple viewpoints can be valid and informative [28]. Caregivers may recognize functional limitations that patients are unaware of or deny. Arguably, assessments of HRQL that are used to inform decision making in health care based on community-based preferences need not be restricted to the patient perspective [29].

It is recommended that parent proxy reports be available for child HRQL measures, a condition that the PedsQL™ also fulfils. The availability of proxy-reports in addition to child reports may be more desirable than reports from the child alone. In medical consultations, the clinicians often look to the parents for guidance regarding medical decision making in relation to their children [16]. The parent view of their child's HRQL can be informative and provide an alternative viewpoint [28].

Further studies are needed to determine the accuracy of the MMQL-UK (PF) proxy reports compared with the MMQL-UK (CF) child reports in other conditions and different age groups of children. The usefulness of the MMQL-UK (PF) in assessing longitudinal outcomes of children also warrants further attention.

## Conclusion

This study established the reliability and validity of a new MMQL proxy report (MMQL-UK (PF)). The MMQL-UK (PF) showed good correlations with the child form (MMQL-UK (CF)) in healthy children, those in public care and those suffering from chronic conditions.

## Abbreviations

Health related quality of life (HRQL), Minneapolis-Manchester Quality of Life Instrument (MMQL), Child Form (CF), Youth Form (YF), Parent Form (PF), Inflammatory Bowel Disease (IBD), Bone Marrow Transplant (BMT), Acute Lymphoblastic Leukaemia (ALL), Multi-centre Research Ethics Committee (MREC), Statistical Package for Social Sciences (SPSS), United Kingdom (UK)

## Competing interests

The author(s) declare that they have no competing interests.

## Authors' contributions

HAH was the primary author on the manuscript and was responsible for methodological and statistical support throughout the study and analysis of the final study results. JGW secured funding and led the study, provided methodological support throughout and was involved in writing the manuscript. PU was the primary researcher, was responsible for co-ordinating and managing the study on a day-to-day basis, for data collection and collation, data analysis and input into writing the manuscript. WYC provided methodological and statistical support throughout the study, was involved in the final study analysis and was involved in writing the manuscript. AM provided clinical support for the study, was involved in designing the study and was involved in writing the manuscript. CE was involved in designing the study, provided methodological support throughout and was involved in writing the manuscript. SJ was involved in designing the study and was involved in writing the manuscript. MEM Jenney provided clinical support, was involved in designing the study, provided methodological support throughout and was involved with writing the manuscript. ITR provided methodological and statistical support throughout the study. All authors read and approved the final manuscript.
